# Assessing Pretreatment Effectiveness for Particulate, Organic and Biological Fouling in a Full-Scale SWRO Desalination Plant

**DOI:** 10.3390/membranes11030167

**Published:** 2021-02-27

**Authors:** Almotasembellah Abushaban, Sergio G. Salinas-Rodriguez, Delia Pastorelli, Jan C. Schippers, Subhanjan Mondal, Said Goueli, Maria D. Kennedy

**Affiliations:** 1 Water Management Department, Faculty of Civil Engineering and Geosciences, Delft University of Technology, Stevinweg 1, 2628 CN Delft, The Netherlands; M.kennedy@un-ihe.org; 2 Environmental Engineering and Water Technology Department, IHE Delft Institute for Water Education, Westvest 7, 2611 AX Delft, The Netherlands; s.salinas@un-ihe.org (S.G.S.-R.); jancschippers@gmail.com (J.C.S.); 3 The International Water Research Institute, Mohammed VI Polytechnic University, Lot 660, Hay Moulay Rachid, Ben Guerir 43150, Morocco; 4 SUEZ International, 183 Ave du 18 Juin 1940, 92500 Rueil-Malmaison, France; delia.pastorelli@suez.com; 5 Promega Corporation, 2800 Woods Hollow Road, Madison, WI 53711, USA; Subhanjan.Mondal@promega.com (S.M.); said.goueli@promega.com (S.G.)

**Keywords:** desalination, fouling potential, seawater reverse osmosis, pretreatment, seawater monitoring

## Abstract

In this study, the removal of particulate, organic and biological fouling potential was investigated in the two-stage dual media filtration (DMF) pretreatment of a full-scale seawater reverse osmosis (SWRO) desalination plant. Moreover, the removal of fouling potential in two-stage DMF (DMF pretreatment) was compared with the removal in two-stage DMF installed after dissolved air floatation (DAF) (DAF-DMF pretreatment). For this purpose, the silt density index (SDI), modified fouling index (MFI), bacterial growth potential (BGP), organic fractions and microbial adenosine triphosphate (ATP) were monitored in the pretreatment processes of two full-scale SWRO plants. Particulate fouling potential was well controlled through the two stages of DMF with significant removal of SDI15 (>80%), MFI0.45 (94%) and microbial ATP (>95%). However, lower removal of biological/organic fouling potential (24–41%) was observed due to frequent chlorination (weekly) of the pretreatment, resulting in low biological activity in the DMFs. Therefore, neutralizing chlorine before media filtration is advised, rather than after, as is the current practice in many full-scale SWRO plants. Comparing overall removal in the DAF-DMF pretreatment to that of the DMF pretreatment showed that DAF improved the removal of biological/organic fouling potential, in which the removal of BGP and biopolymers increased by 40% and 16%, respectively. Overall, monitoring ATP and BGP during the pretreatment processes, particularly in DMF, would be beneficial to enhance biological degradation and lower biofouling potential in SWRO feed water.

## 1. Introduction

Membrane fouling is the main operational challenge that seawater reverse osmosis (SWRO) systems experience [1,2]. Membrane fouling reduces membrane permeability, permeate quality and leads to higher operational pressure [3,4]. Membrane fouling can be due to suspended and colloidal particles, organic matter, dissolved nutrients, biomass and sparingly soluble salts. One or more types of fouling can occur simultaneously depending on feed water quality, operating conditions and type of membrane [5]. To mitigate fouling, pretreatment is applied in full-scale SWRO desalination plants, including sedimentation, dissolved air flotation (DAF), granular media filtration (with or without coagulation) and membrane-based pretreatment (such as microfiltration and ultrafiltration) [6,7,8,9,10]. 

The removal efficiency of pretreatment is typically monitored by particulate fouling indices, such as the silt density index (SDI) and the modified fouling index (MFI), as they are the only American Society for Testing and Materials(ASTM) standard methods that estimate the rate at which particulate fouling may occur in Reverse Osmosis (RO) membrane systems [11,12,13]. The SDI_15_ value recommended by membrane manufacturers in RO feed water to avoid particulate fouling in RO membrane systems differs depending on the water source and pretreatment. For instance, DuPont recommends an SDI_15_ value below 5%/min in the SWRO feed water of open intake and generic conventional pretreatment, while an SDI_15_ value below 2.5%/min is recommended for well or open intake with ultrafiltration (UF) [14]. 

Although SDI and MFI are commonly monitored in SWRO plants to control particulate fouling, no standard method exists to monitor and control biological/organic fouling in SWRO systems [13,15,16]. Recently, several methods to monitor organic/biological fouling potential in the SWRO feed water have been developed, such as assimilable organic carbon (AOC) [13,17,18], membrane biofilm formation rate (mBFR) [19,20] and bacterial growth potential (BGP) [21,22,23]. The relationship between these methods and biological/organic fouling in SWRO systems has not yet been defined. However, while a few attempts have been conducted at pilot and full-scale plants [19,24,25], many more SWRO desalination plants with different pretreatment processes still need to be monitored to establish a good correlation. 

The application of the new biological/organic fouling potential methods showed that conventional pretreatment can remove a low percentage of organic matter. Jeong et al. [18] reported the removal of 5 µg-C/L (12%) of AOC in the dual media filtration (DMF) of Perth SWRO desalination plan. However, the AOC concentration measured in the SWRO feed water was higher than its concentration in the raw seawater, most likely due to the addition of acid and antiscalant during treatment. The same observations were also seen by Weinrich et al. [25] at Al Zawarah SWRO plant (Ajman, United Arab Emirates) and Tampa Bay seawater desalination plant (Florida, USA), in which the removal of AOC during the pretreatment was monitored for two months. They reported 28% removal of AOC (from 14 to 8 µg-C/L) in the DMF of Al Zawarah plant in July, while AOC concentration increased from 2 µg-C/L in the DMF feed water to 147 µg-C/L in the DMF filtrate in November due to coagulation. However, Abushaban et al. [22] reported 17% BGP removal in the DAF system (coupled with 0.5 mg-Fe^3+^/L). In addition, Abushaban et al. [22] reported 53% BGP removal through coagulation (3.6 mg-Fe^3+^/L), flocculation and DMF pretreatment of a SWRO desalination plant.

DAF has been coupled with DMF or UF in a few SWRO desalination plants in the Middle East, where frequent algal bloom events are experienced. Abushaban et al. [23] reported 17% removal of BGP in the DAF system (coupled with 0.5 mg-Fe^3+^/L) and the combination of DAF and UF achieved 50% removal of BGP. Guastalli et al. [26] observed very low removal of organic matter in DAF-DMF and DAF-UF, in which the removals of humics, low molecular weight and biopolymers were 2%, 1% and 18% in the DAF-DMF and 8%, 6% and 41% in the DAF-UF.

To enhance the removal of biological/organic fouling potential in the pretreatment, the use of biological filtration as a pretreatment of SWRO systems was suggested [27,28]. In biological filtration, organic matter can be biodegraded or adsorbed on media. Shrestha et al. [29] compared the removal efficiency of three seawater deep-bed biofilters, with granular-activated carbon (GAC), anthracite and sand as the biofilter media and reported high removal efficiencies in the three biofilters in terms of turbidity (around 50%), MFI (50–80%) and hydrophobic organic compounds (94%). In addition, the removal of dissolved organic carbon (DOC) was 41–88% with GAC media, 7–76% with sand media and 3–71% with anthracite media. Naidu et al. [27] studied the organic removal in seawater GAC biofilters and reported more than 95% (from 24.5 µg-C/L to 0.6 ± 0.2 µg-C/L) removal of AOC, more than 78% (from 33.3 to 5.3 ± 1.1 µg-C/L) removal of transparent exopolymer particles (TEP) and more than 60% (from 1.85 mg/L to 0.75 mg/L) removal of DOC.

In this research, the removal of particulate, biological and organic fouling potential in a full-scale SWRO desalination plant with two-stage DMF coupled with inline coagulation pretreatment was monitored. The newly developed adenosine triphosphate (ATP) and ATP-based BGP methods in seawater (presented by Abushaban et al. [23,30]) were employed to monitor media filtration over time and to assess the removal efficiency of the SWRO pretreatment. Additionally, the removal of fouling potential in two-stage DMF pretreatment was compared with the removal in the pretreatment of another full-scale SWRO desalination plant which includes two-stage DMF preceded by DAF (DAF-DMF).

## 2. Materials and Methods

### 2.1. Description of the SWRO Plant

**Figure 1:**
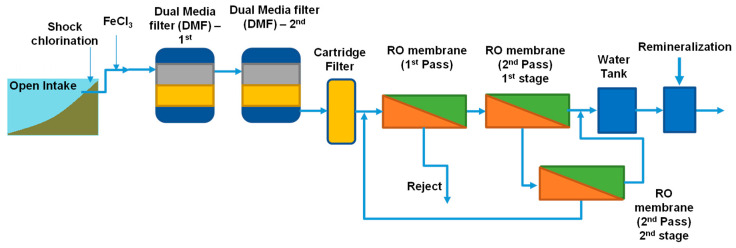
Schematic of the treatment processes of the seawater reverse osmosis (SWRO) desalination plant (plant A).

The study was performed onsite at a full-scale seawater desalination plant located in the Middle East over a period of one week and at the laboratory facilities of IHE Delft in Delft (The Netherlands). Figure 1 shows the treatment scheme of the SWRO plant, which consists of inline coagulation, two stages of DMF, cartridge filtration, RO membrane (two pass) and remineralization.

Seawater is collected through an inlet pipe (open intake) located 1.3 km from the shoreline. Sodium hypochlorite (1 mg-Cl_2_/L) is dosed weekly for 4 h at the intake basin. During chlorination, sodium bi-sulphite is dosed prior to the cartridge filtration to quench the residual chlorine and to prevent oxidation of the RO membranes.

The main pretreatment of the SWRO system is a two-stage DMF operated in series. Table 1 shows the properties of each stage. Prior to the DMF, inline coagulation is applied using 0.7–1.7 mg-Fe^3+^/L. Backwash of the filter is performed using the filtered seawater from the second stage of the DMF. After DMF, seawater is filtered through cartridge filtration (5 µm) to remove any sand and particles coming from the DMF. In addition, 1.9 mg/L of antiscalant is dosed continuously before the cartridge filtration. RO membranes are arranged in a two-pass array. The first RO pass consists of 14 trains operating at 45–57% RO recovery. The second pass consists of seven trains (two stages) of brackish water reverse osmosis (BWRO) operating at 90% RO recovery. The total recovery of the first and second pass is 40–43%.

### 2.2. Sample Collection, Measurement and Transportation

Seawater samples were collected over four days from the seawater intake, after the first stage of DMF, after the second stage of DMF and after cartridge filtration. Chlorination was applied only on the fourth day using a dose of 1 mg-Cl_2_/L. The properties of the collected seawater from the intake, the pretreatment train and potable water are listed in Table 2.

Microbial ATP, BGP, biopolymer concentration, chromatographic DOC, SDI and MFI_0.45_ were measured. Samples were collected in carbon-free bottles for microbial ATP, BGP, chromatographic DOC and biopolymers and measured in triplicate. For SDI and MFI_0.45_ measurement, the samples were collected in 30 L autoclaving bags (Sterilin, Aberbargoed, UK).

One media filter from the first stage of DMF was selected for frequent monitoring. Microbial ATP, BGP, SDI_15_ and MFI_0.45_ of the filtrate of the selected filter were monitored over time (before and after backwashing).

### 2.3. Comparing DMF Pretreatment to DAF-DMF Pretreatment

The removal of fouling potential in the studied full-scale SWRO plant (plant A, Figure 1) was compared with the removal in another full-scale SWRO desalination plant (plant B) presented by Abushaban et al. [24], with the pretreatment of plant B including DAF with 1–5 mg/L of Fe^3+^ and two-stage DMF (with 0.3–1.5 mg/L of Fe^3+^). The location and raw seawater quality of both plants are different. However, the design and operating parameters of the DMFs (both stages) in both plants are identical except for the fact that the DMF of plant B is backwashed with SWRO brine water.

### 2.4. Water Quality Characteristics 

#### 2.4.1. Microbial ATP 

Microbial ATP was measured during the pretreatment of the SWRO using the ATP filtration method, described by Abushaban et al. [30]. Briefly, a seawater sample was filtered through sterile 0.1 µm PVDF membrane filters. The retained microorganisms on the membrane filter surface were rinsed with 2 mL of sterilized artificial seawater water. Then, 5 mL of Water-Glo Lysis reagent (Promega Corp., Madison, WI, USA) was passed through the filter to extract the microbial ATP from the retained microorganisms. Finally, ATP of the filtrate was measured by mixing 100 µL aliquot with 100 µL of ATP Water-Glo detection reagent. The average emitted light measured by the GloMax^®^-20/20 luminometer (Promega Corp., Madison, WI, USA) was converted to microbial ATP concentration based on a calibration curve. Microbial ATP was measured onsite and all samples were collected and measured in triplicate. In total, 88 samples were collected for ATP analysis.

#### 2.4.2. BGP Measurement

BGP measurement indicates the ability of bacteria to grow using the nutrients available in a seawater sample. BGP was measured using the method described by Abushaban et al. [23]. Seawater samples were pasteurized in the plant’s laboratory for 30 min to inactivate microorganisms and thereafter shipped to IHE Delft, the Netherlands, for analysis. Each pasteurized sample was distributed in triplicate into 30 mL carbon-free vials and each vial was inoculated with 10,000 cells/mL (intact cell concentration measured by flow cytometry) using an indigenous microbial consortium from the raw seawater. Seawater samples were incubated at 30 °C and bacterial growth was monitored using microbial ATP in seawater for five days.

#### 2.4.3. Organic Fractions

Biopolymers and chromatographic DOC were detected using liquid chromatography–organic carbon detection (LC-OCD). Seawater samples were shipped in a cool box (5 °C) to DOC-Labor Dr. Huber lab in Germany for analysis.

#### 2.4.4. SDI and MFI_0.45_

The two ASTM methods for particulate fouling potential in RO systems were used (namely SDI and MFI_0.45_). SDI and MFI_0.45_ were measured using a portable SDI/MFI Analyzer (Convergence, Enschede, The Netherlands). It should be noted that the reported value of SDI_15_ should not exceed 75% of the maximum value (6.67%/min) [31]. In cases of high particulate fouling potential, a shorter time needs to be used for the SDI test, such as 10 min (SDI_10_) or 5 min (SDI_5_). If the reported value exceeds 75% of SDI_5_ (maximum value = 15%/min), another test should be used, such as MFI_0.45_ [31]. For this purpose, SDI_5_ was measured in the seawater intake whereas SDI_15_ was measured during the pretreatment (after the DMF first stage, DMF second stage and cartridge filtration (CF)). 

## 3. Results and Discussion

### 3.1. Seawater Intake Water Quality

High daily variation in the seawater quality of the intake was observed within four days (Table 3). The highest chlorophyll a concentration, microbial ATP concentration and MFI_0.45_ were observed on day 3, indicating higher algal concentration in the seawater intake. The highest turbidity (2.8 NTU) was measured on day 4 when chlorination was performed. However, the measured turbidity and MFI_0.45_ in the raw seawater of this plant is low compared to the reported values in raw seawater [32,33].

### 3.2. Particulate Parameters

#### 3.2.1. Silt Density Index (SDI)

**Figure 2:**
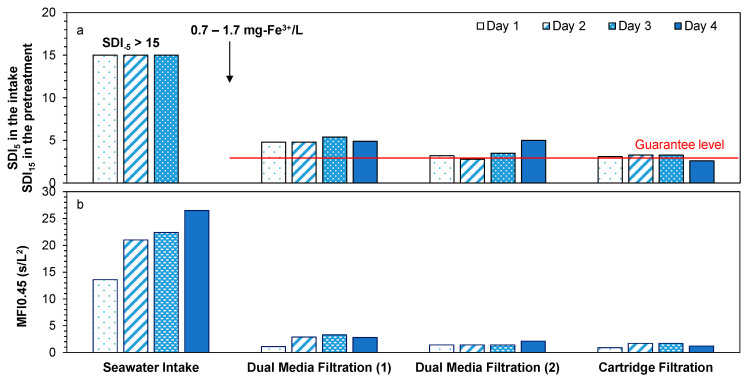
(**a**) Silt density index of 5 min (seawater intake) and 15 min (other samples) and (**b**) modified fouling index of 0.45 µm for the SWRO pretreatment processes of the SWRO desalination plant. Chlorination was applied on day 4.

High particulate fouling potential was observed in the seawater intake, as the measured SDI_5_ was higher than the maximum value (SDI_5_ = 15) indicated by the ASTM method [31]. Significant removal of particles was observed in the first stage of DMF, where 0.7–1.7 mg-Fe^3+^/L was dosed (Figure 2a), and the SDI_15_ after the first stage of DMF averaged at 5%/min. The measured SDI_15_ after the first stage of DMF was higher than the values reported by Bonnelye et al. (2004) [34], who reported a value below 3.3%/min after DMF with 1 mg-Fe^3+^/L as inline coagulation. However, another study from the literature reported even higher SDI_15_ (>6.6%/min) after DMF [35]. Further improvement in SDI_15_ was noted in the second stage of the DMF and in the cartridge filtration, with average SDI_15_ of 3.4 and 3.0%/min, respectively. Higher SDI_15_ after the second stage of DMF on day 4 (SDI_15_ = 5) was measured, which could be attributed to the detachment of a biofilm layer present on the media of the second stage of DMF when chlorine was dosed. This was not seen after the first stage of DMF as the backwashing frequency of the first stage of DMF was much higher (daily) compared with the backwashing frequency of the second stage of DMF (>5 days). Thus, biofilm formation in the second stage of DMF was expected to be greater because of lower backwash frequency. It should be noted that the measured SDI_15_ in the SWRO feed water (after cartridge filtration) met the guarantee level of the manufacturer (SDI_15_ < 4) [10,36,37].

#### 3.2.2. Modified Fouling Index (MFI_0.45_)

The MFI_0.45_ of the seawater (intake) ranged between 14 and 26.5 s/L^2^ with an average of 21 s/L^2^ (Figure 2b), which is lower than the MFI_0.45_ reported by Salinas Rodriguez et al. [32] in the North Sea (20–310 s/L^2^). High MFI_0.45_ values were measured in the seawater intake on day 2 to day 4. The high MFI_0.45_ values of day 2 and day 3 could have been due to the high concentration of algae on these days, for which high chlorophyll a concentration was observed at 17.1 and 32.5 µg/L, respectively. Although lower concentrations of chlorophyll a were measured in the seawater intake on day 4, the MFI_0.45_ values were quite high (26.5 and 21.7 s/L^2^) which could be due to chlorination of the seawater source. Chlorination breaks down organic matter (mainly algae) into small fractions [38], which may increase MFI_0.45_.

A significant reduction (88%) in MFI_0.45_ was noticed in the first stage of DMF with the inline coagulation dosage (0.7–1.7 mg-Fe^3+^/L). The MFI_0.45_ declined from 21 to 2.5 s/L^2^ (Figure 2b). This is an extremely low MFI_0.45_ compared to the reported values after DMF in the literature (12–170 s/L^2^) [32]. The high removal of MFI_0.45_ in the first stage of DMF was confirmed by SDI measurements. Further reduction in MFI_0.45_ was achieved in the second stage of DMF and cartridge filtration. MFI_0.45_ ranged from 1.4 to 2.1 s/L^2^ and from 0.9 to 1.7 s/L^2^ after the second stage of DMF and after CF, respectively. Overall, high removal of particulate fouling potential was measured in the pretreatment of the SWRO plant, in which a decrease in MFI_0.45_ from 21 to 1.4 s/L^2^ was achieved.

### 3.3. Biomass Quantification

**Figure 3:**
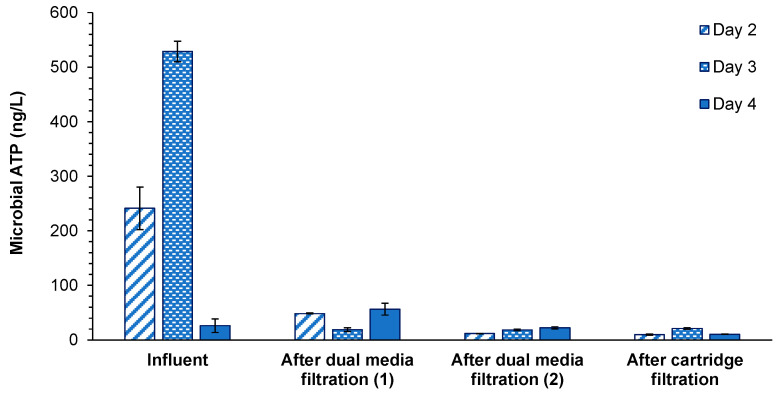
Microbial adenosine triphosphate (ATP) concentrations during the reverse osmosis pretreatment processes of the SWRO desalination plant. Chlorination was applied on day 4.

Microbial ATP in the seawater intake varied from 27 to 525 ng-ATP/L (Figure 3), in which the lowest microbial ATP concentration was observed on day 4 when chlorination was applied in the intake pipe. The highest microbial ATP concentration was observed on day 3, which could be attributed to the algal ATP, as high chlorophyll a concentration was measured on the same day. The measured microbial ATP in the raw seawater was within the range reported in the North Sea (20–1000 ng-ATP/L) [22,30].

Microbial ATP declined significantly (85%), from 385 ng-ATP/L in the intake to less than 60 ng-ATP/L after the first stage of DMF. Abushaban et al. [22] reported 67% removal of microbial ATP through conventional pretreatment (coagulation, flocculation and filtration) in a full-scale SWRO desalination plant, with higher coagulant dose (3.8 mg-Fe^3+^/L) than that applied here (0.7–1.7 mg-Fe^3+^/L). When chlorination was applied (day 4), higher microbial ATP levels were measured after the first stage of DMF compared to the microbial ATP in the seawater intake and after the first stage of DMF in the absence of chlorine, which might be due to the breakdown of the biofilm present on the media of DMF. The same findings were seen for SDI and MFI as well. Further reduction in microbial ATP was observed in the second stage of DMF but no further reduction was observed in the cartridge filtration, as expected. Microbial ATP concentrations ranged between 12 and 22 ng-ATP/L after the second stage of DMF and between 10 and 21 ng-ATP/L after the cartridge filtration. The insignificant reduction in microbial ATP through cartridge filtration was due to the large filter pore size (5 µm) of the cartridge filter compared to marine microorganisms. 

On average, the removal of microorganisms through the SWRO pretreatment was high (95%), and microbial ATP decreased from 385 ng-ATP/L in the seawater intake to 14 ng-ATP/L in the SWRO feed water. The highest removal was observed in the first stage of DMF with inline coagulation (0.7–1.7 mg-Fe^3+^/L). The measured microbial ATP concentration in the SWRO feed is equivalent to 16,000 intact cells/mL (using the correlation reported by Abushaban et al. [30]), which is 1.6 times the concentration of cells used to inoculate BGP samples. This may indicate that faster bacterial growth could occur in the SWRO system if the SWRO feed water contains enough biodegradable organic matter for bacterial growth.

### 3.4. Biological/Organic Fouling Parameters

#### 3.4.1. Organic Fractions

**Figure 4:**
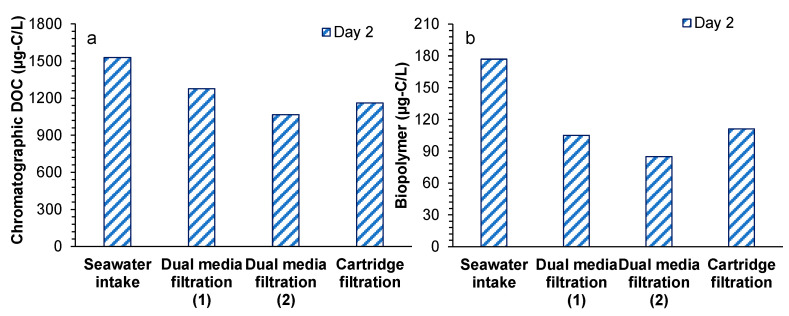
(**a**) Chromatographic dissolved organic carbon and (**b**) biopolymer concentrations during the RO pretreatment processes of the SWRO desalination plant, as measured by LC-OCD.

Chromatographic DOC and biopolymer concentration were measured on day 2 (Figure 4). Chromatographic DOC concentration in the seawater intake was 1.5 mg-C/L and decreased by 16.5% (0.2 mg-C/L) and 16.5% (0.2 mg-C/L) in the first and second stages of DMF. The removal of chromatographic DOC found in the DMFs was close to the removal (0.15 mg-C/L, 15%) reported by Abushaban et al. [23] in the DMF (with 0.8 mg-Fe^3+^/L) of Perth SWRO desalination plant. Higher chromatographic DOC concentration was observed after the cartridge filter, which could be due to the addition of antiscalant [39]. A similar result was also reported by Jeong et al. [18]. A similar trend was observed for the biopolymer concentration, in which the removal of biopolymers in the first stage of DMF (72 µg-C/L, 41%) was higher than the reported removal (10–35 µg-C/L) in literature [18,23,24]. The higher removal of biopolymers could be attributed to the higher coagulation dosage (0.7–1.7 mg-Fe^3+^/L). Overall, the achieved removals of chromatographic DOC and biopolymers during the SWRO pretreatment processes were 24% and 37%, respectively, for which the highest removal was found in the first stage of DMF coupled with inline coagulation.

#### 3.4.2. Bacterial Growth Potential 

**Figure 5:**
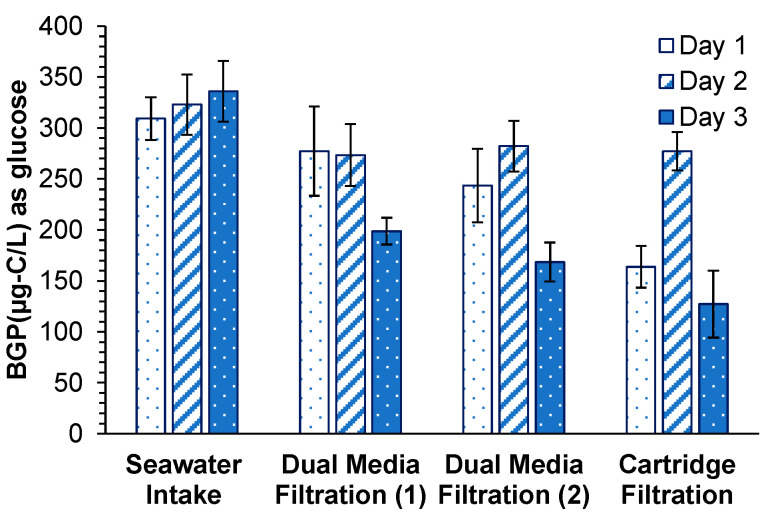
Bacterial growth potential during the RO pretreatment processes of a full-scale SWRO desalination plant.

BGP in the seawater intake ranged between 310 and 340 µg-C/L as glucose (Figure 5). Slight BGP removal was observed in the first stage of DMF (70 µg-C/L as glucose, 22%) and in the second stage of DMF (20 µg-C/L as glucose, 8%). The observed removal of BGP in both stages of DMF was considerably lower than the reported BGP removal in two full-scale desalination plants described by Abushaban et al. [21,23], in which the BGP removal was 55% (190 µg-C/L as glucose) in a gravity DMF coupled with 0.8 mg-Fe^3+^/L and 68% (156 µg-C/L as glucose) in a pressurized DMF with 3.6 mg-Fe^3+^/L. Moreover, Weinrich et al. [40] reported 23–80% removal of AOC (40–280 µg-C/L as acetate) through the sand filtration of Tampa bay desalination plant. The poor removal of BGP in the first and second stages of DMF in this study resulted in SWRO feed water with a high BGP concentration (190 µg-C/L as glucose), despite extensive pretreatment. The level of BGP achieved after the pretreatment (190 µg-C/L as glucose) was close to the BGP (200 µg-C/L as glucose) in the SWRO feed water of a desalination plant (DAF-UF pretreatment) located in the Middle East described by Abushaban et al. [23] where the cleaning-in-place frequency was 6 cleanings in place (CIP’s)/year. The overall BGP removal (41%) was consistent with the observed removal of biopolymers (37%).

### 3.5. Removal Efficiency of Fouling Potential in DMF Pretreatment

Table 4 shows a summary of the measured removal of various fouling indicators and parameters through the pretreatment processes of a full-scale SWRO desalination plant. It can be clearly seen that higher removal rates (>80%) were achieved for particulate fouling indices compared with organic/biological fouling parameters, in which the highest removal was obtained in the first stage of DMF with inline coagulation (0.7–1.7 mg-Fe^3+^/L). The second stage of DMF, followed by cartridge filtration, further improved the seawater quality in terms of particulate fouling potential. The measured SDI_15_ in the SWRO feed water (3%/min) was below the value recommended by the membrane manufacturer [37]. Moreover, using the prediction model for particulate fouling in RO systems described by Salinas Rodriguez et al. [32] and Boerlage et al. [41], particulate fouling should not be expected in the SWRO system within a two-year period as low MFI_0.45_ values were measured in the SWRO feed water (1.4 s/L^2^). Therefore, it can be concluded that two-stage DMF pretreatment in SWRO showed very good removal of particulate fouling potential. Nevertheless, it has been demonstrated that the MFI_0.45_ was not sensitive enough to explain rates of fouling in full-scale RO installations [26,35].

Furthermore, high removal of microbial ATP was observed (97%) in the SWRO pretreatment, with the highest removal reported in the first stage of the DMF (85%). The measured microbial ATP (14 ng-ATP/L~1600 intact cells/mL) in the SWRO feed water may have caused particulate fouling and/or accelerated bacterial growth in the SWRO membrane system and thus may have indirectly increased the rate of biofouling.

Lower removal (24–41%) of organic/biological fouling potential was observed in the SWRO pretreatment compared with the observed removal of particulate fouling and biomass (as microbial ATP). Similar to the particulate fouling potential and microbial ATP, the first stage DMF showed the highest removal of organic/biological fouling potential, including 16.5% of chromatographic DOC (252 µg-C/L), 41% of biopolymers (72 µg-C/L) and 22% of BGP (70 µg-C/L). The low removal of organic/biological fouling potential through the SWRO pretreatment system could be attributed to a low level of biological activity in the media filters. This could have been caused by operational practice such as (i) frequent chlorination of the intake and de-chlorination after the media filtration units, (ii) overly short empty bed contact times in the filter media or (iii) applying backwashing conditions that remove/wash out biofilm layers on filter media, which are required to degrade organic matter (nutrients), preventing further bacterial growth in the downstream SWRO membranes. 

#### Monitoring of DMF First Stage over Time

**Figure 6:**
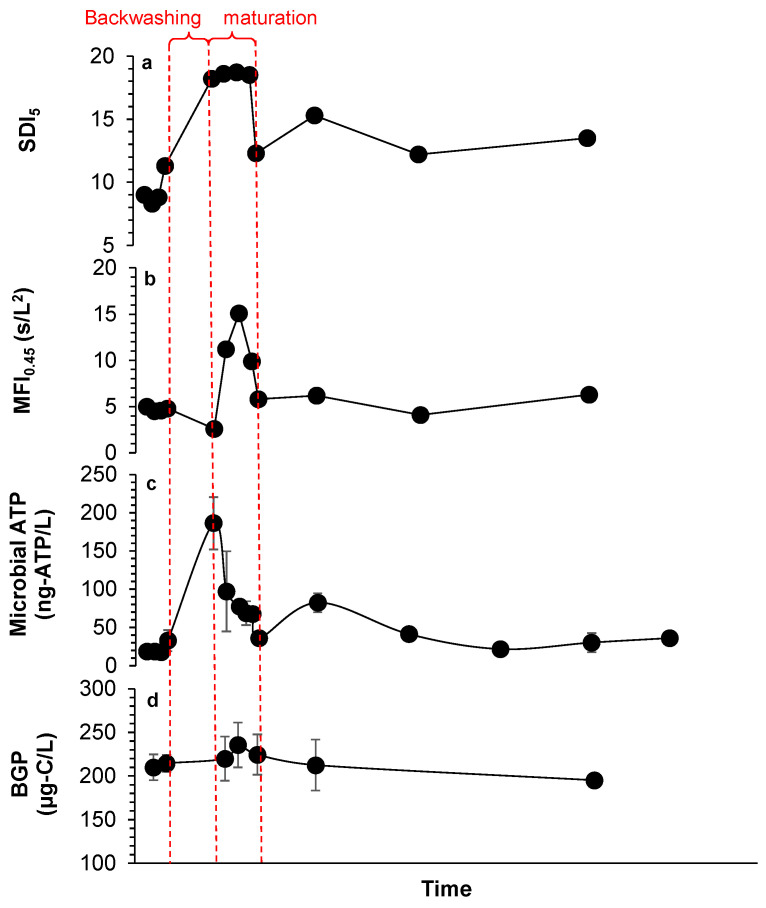
(**a**) SDI_5_, (**b**) MFI_0.45_, (**c**) microbial ATP and (**d**) BGP measured before and after backwashing in the first stage of a dual media filter.

To further investigate the low removal of BGP in DMF, the filtrate of a filter from the first-stage DMF was selected for in-depth monitoring (before and after backwashing), including SDI_5_, MFI_0.45_, microbial ATP and BGP (Figure 6). SDI_5_ and MFI_0.45_ before backwashing were approximately 8%/min and 5 s/L^2^, respectively (Figure 6a,b). High values of SDI_5_ (>15%/min) and MFI_0.45_ (>17 s/L^2^) were measured during the maturation phase, starting immediately after backwashing and with 30 min duration. High SDI_5_ (12–15%/min) and MFI_0.45_ (5–7 s/L^2^) were also measured after maturation, suggesting that the protocol of backwashing needs to be optimized and/or maturation time needs to be prolonged. A similar trend was also observed for microbial ATP (Figure 6c).

An insignificant change in the BGP was found before and after backwashing of the first stage of the DMF (Figure 6d), which could indicate either the absence of a biofilm layer on the media filter or that the biofilm layer attached to the media was not influenced by backwashing (strong biofilm layer), which is unlikely as low removal of BGP was detected in the first stage of the DMF (Figure 5). Moreover, low reduction in BGP was observed over time (10 µg-C/L as glucose within >6 h), indicating poor development of the biofilm on the filter media and thus that it was not a biologically active filter. 

Three options were suggested to explain the low biological activity in the first stage of the DMF: (i) overly harsh backwashing conditions completely removed the biologically active layer, (ii) frequent chlorination destroyed the biofilm, and/or (iii) there was too short an empty bed contact time (EBCT) for biodegradation to occur. Applying overly harsh backwashing conditions is not likely to be the main reason for the low BGP removal, as high values of MFI_0.45_ and SDI_5_ were still measured after maturation of the first stage of the DMF. Moreover, too short an EBCT is not likely to be the cause because higher removal of both BGP and organic matter were measured in another DMF pretreatment system in the full-scale SWRO plant described below (in the following section), with a similar EBCT. Consequently, frequent chlorination of the pretreatment could have been the main reason for the loss in activity of the biofilm layer, as chlorination is applied weekly in the seawater intake [42,43]. Therefore, free chlorine reaches the media filtration and destroys the biofilm layers on the filter media, as discussed earlier. It should be noted that biofilms need a long time to be formed/developed on the filter media (2–7 days) [44]. Thus, to further reduce the BGP of SWRO feed water, priority should be given to (i) reducing the frequency of chlorination in intakes and/or (ii) performing the neutralization step before media filtration, and not after, as is the current practice in many full-scale SWRO plants. Neutralizing chlorine before media filtration supports the development of an active biofilm layer on the media, capable of degrading/removing easily biodegradable organic matter in the feed water. Additionally, longer EBCTs may also significantly improve the biodegradation of organic matter, as well as the removal of biological fouling potential in media filtration. Finally, this result shows that the application of the newly developed ATP and BGP measures to monitor media filtration over time could be beneficial for optimizing SWRO pretreatment. 

### 3.6. Comparing DMF and DAF-DMF Pretreatment

The overall removal achieved through DMF pretreatment (inline coagulation with 0.7–1.7 mg-Fe^3+^/L, two stages of DMF and cartridge filtration) of plant A (described above) was compared to the removal of DAF-DMF (DAF with 1–5 mg-Fe^3+^/L, inline coagulation with 0.3–1.5 mg-Fe^3+^/L, two stages of DMF and cartridge filtration) of plant B. It should be noted that the feed water quality of both plants is different (Table 5). However, the design and operational parameters of the DMFs in both plants (the type, height and size of media, filtration rate and contact time, and the backwash frequency and duration) are similar except that SWRO brine is used to backwash the DMFs in plant B.

A similar overall removal of particulate fouling potential was achieved within the pretreatments of plant A and B. However, particulate fouling potential measured in the raw seawater of plant B (40.5 s/L^2^, as MFI_0.45_) was considerably higher than in plant A (21 s/L^2^, as MFI_0.45_). The highest removal of particulate fouling potential was observed in the first stages of the DMFs of plant A (>65%) and plant B (>68%). The SDI_15_ and MFI_0.45_ after the first stages of the DMFs of plant A and plant B were in the same range. One may conclude that DAF (plant B) did not significantly contribute to removing particulate fouling potential but it could have reduced particle loading in the succeeding DMF and thus increased the filtration rate of DMF. Thus, in the case of plant B, DAF was installed to overcome the high particulate fouling potential in raw seawater. It is worth mentioning that low SDI_15_ and MFI_0.45_ were measured after the DAF-DMF of plant B (3.5%/min and 1.5 s/L^2^, respectively) compared to the values reported by Kim et al. (4.7%/min and 8.5 s/L^2^) [45] after the DMF of a DAF-DMF pilot SWRO plant; however, particulate fouling potential in raw seawater was much lower (26–33 s/L^2^, as MFI_0.45_).

Microbial ATP concentrations in the raw seawater of plant A and plant B were in the same range (Table 5). Even though high removal (48%) of microbial ATP was observed in the DAF of plant B, the overall removal of microbial ATP through the pretreatment of both plants was more or less similar (371 and 335 ng-ATP/L). This may indicate that the addition of DAF did not improve the removal of microbial ATP, as the DAF system is usually used to remove floating particulates, such as algal cells during algal bloom events [10,46].

The biological/organic fouling potential observed in the raw seawater of plant B was approximately 50% higher in terms of BGP, chromatographic DOC and biopolymer concentration than in plant A. Moreover, the actual removal of biological/organic fouling potential in plant B (368 µg/L of BGP, 449 µg/L of chromatographic DOC and 163 µg/L of biopolymers) was significantly higher than in plant A (130 µg/L of BGP, 368 µg/L of chromatographic DOC and 66 µg/L of biopolymers). The additional removal in plant B was mainly attributed to the DAF system, in which 260 µg-C/L of BGP, 109 µg-C/L of chromatographic DOC and 83 µg-C/L of biopolymer were removed due to the higher applied coagulant dose (1–5 mg-Fe^3+^/L). 

To summarize, comparing DMF pretreatment with DAF-DMF pretreatment showed that DAF significantly improved the removal of biological/organic fouling potential in terms of BGP, chromatographic DOC and biopolymer. Although, the overall removal of particulate fouling potential (as MFI_0.45_ and SDI_15_) and microbial ATP in the DAF-DMF pretreatment and DMF pretreatment was comparable, DAF may improve the operation of DMF by decreasing head loss and improving filter backwashing.

## 4. Conclusions

- This study assessed the performance of SWRO pretreatment (DAF, DMF1 and DMF2) with regard to controlling particulate, organic and biological fouling in two full-scale desalination plants.
- The removal of particulate fouling potential (SDI, MFI_0.45_), biological/organic fouling potential (BGP, chromatographic DOC and biopolymer concentration), as well as microbial ATP, was monitored in the pretreatment (two-stage DMF with 0.7–1.7 mg-Fe^3+^/L) of a full-scale SWRO desalination plant.
- High removal (>80%) of particulate fouling potential (in terms of SDI, MFI_0.45_) and microbial ATP was achieved in the pretreatment (inline coagulation and two-stage DMF), in which the highest removal (65–85%) was observed in the first stage of the DMF. However, significantly lower removal of the organic/biofouling potential in terms of BGP, chromatographic DOC and biopolymers was achieved in the first stage of the DMF (22–41%) and after pretreatment (24–41%). This was attributed to frequent (weekly) chlorination of the intake and the fact that de-chlorination takes place after media filtration, resulting in damage to the biofilm layer on the filter media, which was also reflected in the ATP and BGP measurements.
- The application of the newly developed ATP and BGP methods to monitor media filtration over time showed that they can be used to improve current practices in SWRO desalination plants, such as chlorination/de-chlorination and backwashing. Thus, they can be used to optimize the removal of biological/organic fouling potential in the pretreatment.
- The overall removal achieved through two stages of DMF (with 0.7–1.7 mg-Fe^3+^/L) was compared to the removal of DAF (with 1–5 mg-Fe^3+^/L) and two-stage DMF (with 0.3–1.5 mg-Fe^3+^/L) and it was found that DAF significantly improved the removal of biological/organic fouling potential in terms of BGP and biopolymers, by 40% and 16%, respectively.

## Figures and Tables

**Table 1:** Characteristics and operational properties of the two-stage media filtration.

	1st Stage of the DMF	2nd Stage of the DMF
Number and type of filter	23 horizontal pressure filters	16 horizontal pressure filters
Filtration rate	12.5 m/h	19.5 m/h
Media size	0.95 mm sand and 1.5 mm anthracite	0.28 mm sand and 0.95 mm anthracite
Filtration period	~24 h	120 h

DMF: Dual Media Filtration.

**Table 2:** The water properties of the seawater reverse osmosis influent and effluent (potable).

Parameter	Influent	Potable Water
pH	8.0–8.2	8.0–8.3
Conductivity	54 mS/cm	90–400 µS/cm
Temperature	30–34 °C	32–35 °C

**Table 3:** Seawater properties in the intake.

Day	Chlorophyll a (µg/L)	Microbial ATP (ng-ATP/L)	Turbidity (NTU)	SDI_5_ (%/min)	MFI_0.45_ (s/L^2^)
1	10.3	NA	1.1	>15	14.2
2	17.1	240	1.1	>15	21.0
3	32.5	535	0.9	>15	22.4
4	6.9	27	2.8	>15	16.5

ATP: Adenosine Triphosphate, SDI; Sit density index, MFI; Modified fouling index, NA: data is not available.

**Table 4:** Summary of removal of different fouling parameters in SWRO pretreatment.

Parameter	Removal in DMF1	Removal in DMF2	Removal in CF	Overall Removal
SDI, %/min (%)	>10 (>65%)	1.6 (32%)	0.4 (12%)	>12 (>80%)
MFI_0.45_, s/L^2^ (%)	18.5 (88%)	0.9 (36%)	0.2 (12%)	19.6 (94%)
Microbial ATP, ng-ATP/L (%)	325 (85%)	43 (72%)	3 (18%)	371 (97%)
BGP, µg-C/L (%)	70 (22%)	20 (8%)	40 (17%)	130 (41%)
Chromatographic DOC, µg-C/L (%)	252 (17%)	209 (17%)	−93 (−9%)	368 (24%)
Biopolymers, µg-C/L (%)	72 (41%)	20 (24%)	−26 (−31%)	66 (37%)

CF: Cartridge Filtration, BGP: Bacterial growth potential, DOC; Dissolved organic carbon.

**Table 5:** Comparing dual media filtration (DMF) pretreatment (plant A) to dissolved air floatation followed with dual media filtration (DAF-DMF) pretreatment (plant B).

Parameter	Plant	Raw Seawater	DAF	DMF1	DMF2	CF	Overall Removal
SDI_15_, %/min (% removal)	A	>15 *	-	5 (>65%)	3.4 (32%)	3.0 (12%)	>12 (>80%)
	B	>15 *	NA	4.8 (>68%)	3.9 (19%)	3.5 (10%)	>11.5 (>76%)
MFI_0.45_, s/L^2^ (% removal)	A	21	-	2.5 (88%)	1.6 (36%)	1.4 (12%)	19.6 (93.5%)
	B	40.5	NA	3.6 (91%)	1.7 (52%)	1.3 (24%)	39.2 (97%)
Microbial ATP, ng-ATP/L (% removal)	A	385	-	60 (85%)	17 (72%)	14 (18%)	371 (97%)
	B	370	191 (48%)	85.5 (55%)	42.5 (50%)	35 (18%)	335 (91%)
BGP, µg-C/L (% removal)	A	320	-	250 (22%)	230 (8%)	190 (17%)	130 (41%)
	B	460	200 (57%)	120 (40%)	107 (11%)	92 (14%)	368 (80%)
Chromatographic DOC, µg-C/L (% removal)	A	1528	-	1276 (17%)	1067 (17%)	1160 (−9%)	368 (24%)
	B	2015	1904 (6%)	1588 (11%)	1590 (0%)	1566 (1.5%)	449 (22%)
Biopolymers, µg-C/L (% removal)	A	177	-	105 (41%)	85 (24%)	111 (−31%)	66 (37%)
	B	311	228 (27%)	194 (15%)	151 (22%)	148 (2%)	163 (53%)

* Measured as SDI_5_.

## Data Availability

Not applicable.
